# A Comparison of Capacitive Deionization and Membrane Capacitive Deionization Using Novel Fabricated Ion Exchange Membranes

**DOI:** 10.3390/ma16134872

**Published:** 2023-07-07

**Authors:** Mahmoud M. Elewa, Mervette El Batouti, Nouf F. Al-Harby

**Affiliations:** 1 Arab Academy for Science, Technology and Maritime Transport, Alexandria P.O. Box 1029, Egypt; mahmoud.elewa@aast.edu; 2 Chemistry Department, Faculty of Science, Alexandria University, Alexandria 21526, Egypt; mervette_b@yahoo.com; 3 Department of Chemistry, College of Science, Qassim University, Buraydah 51452, Saudi Arabia

**Keywords:** water, desalination, capacitive deionization, membrane capacitive deionization, charge efficiency, ion exchange membranes

## Abstract

Another technique for desalination, known as membrane capacitive deionization (MCDI), has been investigated as an alternative. This approach has the potential to lower the voltage that is required, in addition to improving the ability to renew the electrodes. In this study, the desalination effectiveness of capacitive deionization (CDI) was compared to that of MCDI, employing newly produced cellulose acetate ion exchange membranes (IEMs), which were utilized for the very first time in MCDI. As expected, the salt adsorption and charge efficiency of MCDI were shown to be higher than those of CDI. Despite this, the unique electrosorption behavior of the former reveals that ion transport via the IEMs is a crucial rate-controlling step in the desalination process. We monitored the concentration of salt in the CDI and MCDI effluent streams, but we also evaluated the pH of the effluent stream in each of these systems and investigated the factors that may have caused these shifts. The significant change in pH that takes place during one adsorption and desorption cycle in CDI (pH range: 2.3–11.6) may cause problems in feed water that already contains components that are prone to scaling. In the case of MCDI, the fall in pH was only slightly more noticeable. Based on these findings, it appears that CDI and MCDI are promising new desalination techniques that has the potential to be more ecologically friendly and efficient than conventional methods of desalination. MCDI has some advantages over CDI in its higher salt removal efficiency, faster regeneration, and longer lifetime, but it is also more expensive and complex. The best choice for a particular application will depend on the specific requirements.

## 1. Introduction

Emerging technologies, like forward osmosis (FO) [1], membrane distillation (MD) [2,3], pervaporation (PV) [4,5], capacitive deionization (CDI) [6,7,8], electrodialysis (ED) [9,10], electrodialysis reversal (EDR) [11], and microbial desalination cells (MDC) [12,13], have lower energy consumption and lower environmental impacts than conventional methods [14,15]. Capacitive deionization (CDI) is the most appealing of these techniques due to its great energy efficiency, lack of reliance on high-pressure pumps, environmental friendliness, ease of operation, and low energy consumption [16,17,18,19]. Furthermore, it may be incorporated with solar energy or any other renewable energy source in remote places with no environmental repercussions [20].

CDI is an electrochemical method for desalinating brackish water that has the added benefit of coupling the rejection of salt with the storage of electrochemical energy [21,22,23]. The CDI process involves the passage of influent seawater between two polarized porous carbon electrodes to produce counter-ion adsorption inside electrical double layers (EDL), which removes salt from the bulk solution. To regenerate adsorption capacity, the energy that has been stored in the electrodes must be discharged by switching the direction of the current flow. This results in the salt that has been adsorbed being released into the brine stream. The salt adsorption capacity (SAC), and the energy-normalized adsorption of salt (ENAS), are directly related to the charge efficiency (CE) of a CDI cell, which is defined as the ratio of adsorbed charge to the applied charge. A CDI cell’s charge efficiency (CE) is arguably the most important cell performance metric [23,24,25].

**Figure 1:**
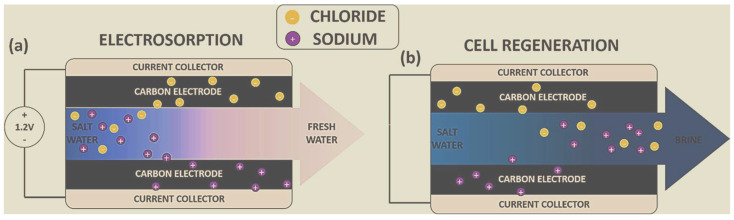
CDI cell under (**a**) electrosorption and (**b**) regeneration steps [32].

A CDI unit comprises two porous electrodes (often carbon electrodes) and a separator (either a porous dielectric material or an open channel) [26]. Typically, the electrodes are treated to a potential difference of 1–1.4 V [27]. The saline or contaminated water passes between the positive and negative electrodes, where the unwanted ions in the water move into an electric double layer (EDL) along the electrode–water interface, thereby removing the unwanted salts or contaminants [28] via electrosorption on the electrode surfaces (Figure 1a). During the discharging step “desorption process” [29], the ions are trapped electrostatically at the electrode surface and then released by reversing the polarity. (Figure 1b). CDI unit can complete countless adsorption–desorption cycles. The deterioration of the electrodes will limit the lifespan of the cell. Oxygen and some organic molecules contained in water may destroy electrodes by oxidation [30], hence lowering the electrosorption capacity and the lifespan of the cell [31,32].

The fabrication of composite CDI electrodes with water-soluble polymers that are cross-linked should reduce chemical use and enhance performance. Most CDI electrodes use a hydrophobic polymer, such as polyvinylidene fluoride (PVDF), to bind activated carbon sheets in aqueous conditions, whereas PDVF necessitates the incorporation of organic solvents into the active material matrix [22].

In addition, the saturated electrode regeneration process is carried out by short circuits or reversed polarity, which results in sporadic functioning and decreased flow efficiency [33,34]. Alternatives to conventional CDI systems that overcome these drawbacks include flow electrode capacitive deionization (FCDI). Conductive slurry-type electrodes, made up of carbon particles with a high specific surface area, that are suspended in the electrolyte and may be recirculated throughout the desalination, are used in FCDI in lieu of the porous electrodes that remain stationary in CDI cells [35].

By inserting complementary fixed-charge groups at the diffusion interface of the electrode or directly onto the carbon surface, charge efficiency may be enhanced. Ion exchange membranes (IEMs) are positioned between the electrodes and the desalination channel in the process known as membrane capacitive deionization (MCDI). IEMs are commonly constructed out of polymeric materials, which, as a result of the presence of covalently linked groups in the backbone, have a high density of fixed charge carriers. For example, quaternary ammonium cations (NH_4_^+^) may be found in anion exchange membranes (AEMs), while sulfonate (SO_3_^−^) and phosphate (PO_4_^−3^) groups can be found in cation exchange membranes (CEMs). These electrically charged species may either be found in the polymers in their native state or be grafted onto the membrane via the use of chemical processes [36,37] The purpose of this placement is to increase the counter-ion flux and prevent co-ions that are discharged from EDLs during charging from entering the desalination flow channel [38]. IEMs have been shown to improve charge efficiency [39,40,41,42,43,44,45,46,47,48]; they also prevent oxygen diffusion [49,50,51]. Ion-exchange materials, either in the form of directly cast IEMs [38,43,52] or polyelectrolyte layers, have been added to the surface of the CDI electrode to lower the electrical resistance and the associated chemical cost [53]. However, this results in a decrease in the charge density of the polymer coating, which has a detrimental influence on both the retention of co-ions and the charge efficiency [52,53]. Immobilizing chemical charges on the surface of the carbon may also inhibit co-ion repulsion. This alters the charge balance in the micropores and encourages counter-ion adsorption inside the electric double layer [54,55,56]. Fixing charge on the carbon surface and covering the electrode with IEMs enhances performance, but this methodological dependence on strong acids and organic solvents also drives up system costs and the potential for global warming [57].

In membrane-based electric desalination systems, ion transport across ion exchange membranes (IEMs) causes ion concentration polarisation (ICP) events owing to the ionic mismatch at the interface between the IEMs and bulk electrolytes [58]. On both sides of the IEM, ion concentration gradients arise, generating an ion enrichment layer and an ion depletion layer. Han et al. abandoned the anion exchange membrane (AEM) and created ICP desalination using just a cation exchange membrane (CEM) to increase salt removal and energy efficiency [58,59]. ICP is a unipolar desalination process with a single kind of IEM that pushes the limits of energy efficiency optimization [60]. The ion depletion layer has been carefully researched in the majority of electro-membrane processes (e.g., ED and ICP) since it is related to a trade-off between salt removal rate and energy efficiency [59,61]. On the other hand, the ion enrichment layer is closely connected to the brine concentration ratio and enables ion transport. However, research on the impacts of the ion enrichment layer on desalination systems is currently insufficient.

When the electric field potential is greater than 1.23 V or 1.36 V, respectively, anodic oxidation of water and chloride ions may take place in the CDI cell. To prevent this, a low voltage is given to the cell. Equations (1) and (2) explain the well-known oxidation of water that takes place when the applied potential is higher than 1.23 V [62,63,64]. This oxidation takes place when oxygen is added to the water. Both equations result in the release of protons, which lowers the pH of the water, turning it into an acidic state that is unhealthy for human consumption.

(1) $$2H_{2}O\to O_{2}+4H^{+}+4e^{-}\;\;E^{o}=1.23\;V$$

(2) $$H_{2}O\to {\mathrm{HO}}^{*}+H^{+}+e^{-}\;\;\;E^{o}=2.80\;V$$

The following series of events is involved in the anodic oxidation of Cl^−^, which takes place when the applied potential is greater than 1.36 V [62,65]. Hydrolysis and the produced disproportionation form hypochlorous acid HClO (Equation (4)), and lead to the formation of chlorate ClO_3_^−^ due to the anodic oxidation of HClO (Equation (5))which leads to the formation of chlorine gas (due to the oxidation reactions of Cl^−^ ions at the anode; (Equation (3)). Hydrolysis and disproportionation of the product follows (Equation (5)):

(3) $$2{\mathrm{Cl}}^{-}\to {\mathrm{Cl}}_{2}+2e^{-}\;\;E^{o}=1.36\;V$$

(4) $${\mathrm{Cl}}_{2}+H_{2}O\to \mathrm{HClO}+H^{+}+{\mathrm{Cl}}^{-}$$

(5) $$6\mathrm{HClO}+{3H}_{2}O\to {2\mathrm{ClO}}_{3}^{-}+4{\mathrm{Cl}}^{-}+3/2O_{2}+12H^{+}+6e^{-}$$

Miniature solar cells can power capacitive deionization (CDI) devices since desalination requires a low voltage. This is especially useful in many Middle East and North Africa (MENA) areas without electricity. Electrochemical systems like capacitive deionization (CDI) remove tiny ions from water, unlike RO. Table 1 compares energy needs for desalination technologies. Three desalination methods exist. This category includes thermal de-salination methods like multi-stage flash distillation (MSF) and multiple effect distillation (MED), membrane methods like reverse osmosis (RO) and electrodialysis (ED), and electrochemical methods like capacitive deionization (CDI). The operating principle and water ionic strength affect desalination equipment energy consumption. The thermal methods evaporate salty water, condense it, and collect fresh water. The evaporating and condensing of water require a lot of energy. Desalination companies invested much in membrane technology research to reduce energy use. Because of this, membrane desalination now accounts for 60% of worldwide desalinated freshwater output [66]. Membrane-based desalination methods are market leaders and 80% of desalination facilities are membrane plants. However, membrane desalination is mostly used to desalinate saltwater since brackish water desalination is too expensive [67]. Desalination procedures require a lot of energy for water pumping and membrane performance. In low-salinity feed streams, capacitive deionization (CDI) uses less energy than other current processes [68]. Thus, it suits socio-economically deprived societies like those in the MENA.

Membrane fouling during lengthy operations prohibits MCDI from being scaled up and used in many applications. Raw feed’s high fouling potential has plagued the food industry, especially wastewater treatment. Microorganisms, colloidal particles, and organic and inorganic compounds, present in high concentrations in wastewater and food waste streams, can severely contaminate CEMs. CEMs may lose perm-selectivity, ion exchange capacity, and electrical resistance due to salt and organic matter deposition on their surfaces. More importantly, it will limit the CEMs’ lifespan and reuse capacity while raising running costs. A membrane with excellent antifouling capabilities improves its application and durability. Handling complex water that might clog the membrane requires this. Cleaning better removes membrane fouling. Water rinsing, alkali, acid, water–ethanol combinations, and surfactant/enzymatic solution cleaning have been studied. In situ cleaning methods have also been studied. Before choosing the best cleaning method, consider the membranes, foulants, and application. Alkaline cleansing periodically removes organic foulants. However, salt scaling requires acid cleaning. In food applications, surfactants and water–ethanol combinations are the most effective foulant removal methods [3,75,76].

CDI and MCDI extract ions from water using electrical charge and ion-selective membranes. The step-by-step CDI/MCDI mechanism is as follows: (1) set-up: anodes and cathodes on each side of ion-exchange membranes make up a standard CDI/MCDI configuration. MCDI has extra ion-selective membranes between the centre and anode and cathode compartments. (2) Charge stage: (a) feedwater flow: ionised saline feedwater flows through the middle compartment between the ion-exchange membranes. (b) Voltage application: voltage between the electrodes creates an electric field in the centre compartment. (3) Ion adsorption: (a) electrostatic attraction: the applied electric field attracts feedwater ions to charged electrodes. (b) Ion adsorption: the oppositely charged electrodes attract feedwater ions and adsorbed them. (4) Ion removal: (a) deionization process: ions adsorbed onto the electrodes’ lower ion concentration in the centre compartment, deionizing feedwater. (b) Ion migration: during charging, cations migrate to the anode and anions to the cathode. Ion-selective membranes in MCDI aid migration. (c) Ion-selective membrane function: in MCDI, ion-selective membranes enable certain ions to flow while blocking others, separating cations and anions. (5) Discharge stage: (a) flushing the electrodes: after ion removal, the voltage is switched off and the electrodes are flushed to remove adsorbed ions. (b) Regeneration: flushed ions are usually sent to a waste stream or regenerated for recovery or treatment. (6) Repeat process: reapplying voltage to the electrodes repeats the CDI/MCDI cycle, continuing desalination.

The objective of the present work is to evaluate and compare the desalination of simulated brackish water using CDI and MDCI techniques. A specially devised plexiglass cell was tested for the desalination of simulated brackish water at different concentrations. Both electrodes were prepared in the laboratory with PVDF, to bind activated carbon sheets. The innovative IEMs were selected as the best results out of 25 prepared membranes from our previous work [68] and used for the first time in MCDI. The variables investigated were batch vs. single-pass (SP) mode of operation, CDI vs. MCDI (SP), changes in treated water conductivity with cell potential (SP), and the effect of pH.

## 2. Materials and Methods

### 2.1. Materials

All compounds used were analytical grade. Electrode manufacture using activated carbon (AC Norit SA 4, Cabot Norit Activated Carbon, Marshall, TX, USA), polyvinylidene fluoride (PVDF, Sigma-Aldrich, Waltham, MA, USA), N-N dimethylformamide (DMF, 99.8%, Merck Millipore, Burlington, MA, USA), graphite sheet (DSN 530, Suzhou Dasen Electronics Material Co., Suzhou, China), cellulose acetate (CA, powder from Sigma, USA, acetyl content 39.8%; average mol wt ~ 30,000), Amberlite 252 Na (strong macroporous cation-exchange resin, styrene-divinylbenzene copolymer matrix from Lenntech, Delfgauw, The Netherlands), Amberlite IRA900 Cl (strong macroporous anion-exchange resin, styrene-divinylbenzene copolymer matrix from Lenntech), acetone (mol wt 58.08 g/mol from Adwic Laboratory Chemicals, Cairo, Egypt), and dimethyl phthalate (DMF, mol wt 194.18 g/mol from Koch-Light Laboratories Ltd., Haverhill, UK). Electrolyte solutions were typically made with 99.7 per cent pure sodium chloride (NaCl). Purified water was used to make the solutions.

### 2.2. Electrode Fabrication Method

As a part of making the porous carbon electrodes, PVDF was dissolved in DMF at 105 °C. The mixture was then supplemented with activated carbon powder at a mass ratio of 1:10 AC to PVDF. The thick slurry was cast on a graphite sheet (DSN 530, Suzhou Dasen Electronics Material Co., Suzhou, China) as a current collector after 2 h of mixing and evaporation of the surplus solvent. Overnight, the electrode was dried out in a 105 °C oven with a fan and, the next day, it was baked in an 80 °C vacuum oven for 2 h.

### 2.3. Ion Exchange Membranes Fabrication

IEMs were prepared using the phase inversion method, at room temperature (25 ± 2 °C), in which the cellulose esters constituted the polymer matrix, and the various ion-exchange resins were mixed within the polymer to provide the heterogeneous AEM and CEM. This process was carried out while the IEMs were kept at a temperature of 25 ± 2 °C. Using an accurate analytical balance, a known weight of cellulose ester was weighed. Next, a mixture of solvents in specified quantities was added to the ester in a wide-mouthed bottle with a tight sintered glass cover. This was followed by the addition of the desired resin, whether in ground form or intact. Finally, the whole thing was thoroughly mixed with a thick glass rod until a thick homogeneous transparent solution was obtained, with the resin evenly dispersed in the viscous matrix. After pouring a suitable amount into the boat “container or support structure that holds the casting solution or material during the membrane casting process, facilitating the formation of a thin membrane or film”, which was equipped with an inclined doctor’s blade fitted at the necessary height above the plate, the viscus solution was then cast on a glass plate using a manual casting assembly. The plate was placed at the desired height above the plate. The boat was then moved slowly at a constant speed during which a uniform membrane was formed on the glass plate, and the as-cast membrane was allowed to evaporate for exactly 0.5 min. The as-cast membrane was then immersed in distilled water and left for at least 1 h, during which time membrane coagulation took place. After the coagulation process was finished, the membrane may be readily withdrawn from the water bath and steeped in a new bath of freshly distilled water until it was ready to be used. This was because the membrane progressively peels away from the glass plate as the coagulation process progresses. De-esterification can also be accomplished with the membrane by first soaking it in a solution that contains 1% NaOH and 20% NaCl in distilled water, then covering the container and letting it sit for 24 h. After that, the membrane should be thoroughly rinsed with more distilled water, and then it should be stored in distilled water, ready for use.

### 2.4. The Characterisation of IEMs

To characterise the membranes that were fabricated, they were put through four tests: measurement of ion-exchange capacity, determination of % swelling, determination of perm-selectivity, and determination of membrane thickness.

#### 2.4.1. The Ion-Exchange Capacity (IEC)

IEC is quantified as the quantity of milliequivalent of ion-exchange groups present in a 1-gram dry membrane (meq/g dry membrane), according to a previously established definition [77]. The ion-exchange capacity (IEC) of a charged membrane can be determined through a process of titration of fixed ions, such as SO_3_^−^ or R_4_N^+^ groups, using 1 N NaOH or 1 N HCl. To perform this process, a sample of the CEM or AEM is first weighed and equilibrated for 1 h in either 1 N HCl or 1 N NaOH. The sample is then back-titrated with NaOH or HCl, using phenolphthalein as an indicator. Finally, the sample is removed from the titration solution and thoroughly rinsed. Subsequent to drying the membrane at a temperature of 30 °C for a duration of 24 h, the ion exchange capacity (IEC) is computed for the dehydrated membrane. The ion-exchange capacity determination was carried out using the titration procedure, which was repeated four times. The resulting data were reported as means accompanied by their respective standard deviations.

(6) $$IEC(mmol/g)=\frac{{Titre\;value}_{\left(mL\right)}-{Normality}_{\left(NaOH\;or\;HCl\right)}}{W_{d}}$$

The variable *W_d_* represents the weight of the polymer membrane in its dry state, measured in grams.

#### 2.4.2. The Percentage of Swelling

The % swelling was determined by subjecting the wet membrane to a process of surface drying using highly absorbent paper, followed by weighing the membrane on an analytical balance to the fourth decimal place. The membrane was then allowed to air dry for a minimum of 24 h at a temperature of 30 °C, after which it was weighed again until a constant weight was achieved. The measurements were conducted in triplicate and the outcomes were reported as the mean value along with the standard deviation. The percentage of swelling was subsequently calculated using Equation (7):

(7) $$\%\;swelling=\left[\frac{\left(W_{w}-W_{d}\right)}{W_{w}}\right]\times 100$$

The variable *W_w_* represents the wet weight of the membrane sample, measured in grammes.

#### 2.4.3. Determination of Perm-Selectivity for Membranes

The term “perm-selectivity” pertains to the capacity of a membrane to differentiate between positively charged cations and negatively charged anions. The measurement of concentration potential can be conducted by dividing the solutions of the same electrolyte at different concentrations by the test membrane. Potassium chloride is frequently employed as an electrolyte due to the comparable mobility of its constituent potassium and chloride ions [38]. The maximum potential generated by an ideal selective separator can be described by an equation that is related to the Donnan equation. The formula for P is derived as follows: P equals (RT divided by F) multiplied by the natural logarithm of the ratio of C1 to C2. In this equation, R represents the gas constant, T represents the absolute temperature, F represents Faraday’s constant, and C1 and C2 represent the concentrations of the electrolyte. The theoretical potential of 58 millivolts can be observed for a concentration ratio of 10:1. The calculation of perm-selectivity involves determining the estimated potential as a proportion of the theoretical potential of a separator with 100% selectivity. For the test to yield precise results, it is imperative that the two electrodes exhibit parity. The measurement of potential between two electrodes immersed in a common electrolyte can be conducted. As an alternative approach, it is possible to interchange the electrodes subsequent to the computation, and subsequently determine the mean potential value [38]. The perm-selectivity is contingent upon the concentration of the electrolyte, rather than the thickness of the membrane. This observation aligns precisely with the predictions posited by the Donnan hypothesis.

#### 2.4.4. The Fourier Transform Infrared (FTIR)

FTIR analysis of the membrane was conducted using a Fourier transform infrared spectroscopy instrument (VERTEX 70, Bruker Co., Karlsruhe, Germany).

#### 2.4.5. Water Contact Angle

The water contact angle was determined using a contact angle goniometer (JC-2000C Contact Angle Metre, Powereach Co., Shanghai, China).

### 2.5. Capacitive Deionization Set-Up

**Figure 2:**
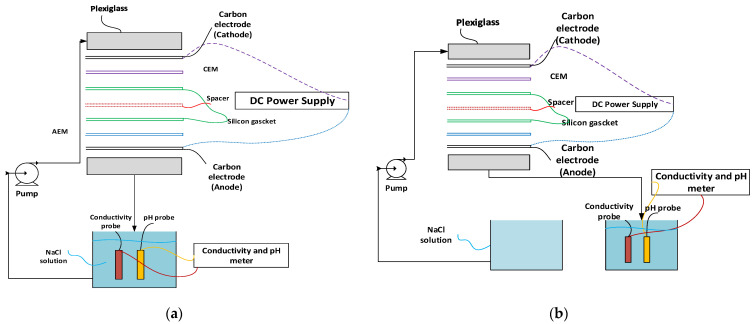
Capacitive Deionization cell construction: (**a**) batch and (**b**) single-pass mode of operation.

Plexiglass sheets with a thickness of 5 millimetres are used to construct the cell. Two electrodes create the capacitive deionization cell. Each electrode has an average thickness of 170 ± 20 µm and a carbon mass of 7.5 ± 0.5 mg/cm^2^. Before the desalination test, the carbon electrodes were activated with three repeated polarization cycles, first by applying a voltage of 1.2 V over 10 min for the charging step, and then 0 V over 10 min for the discharging step. As an MCDI cell, cation and anion exchange membranes were explained in detail in our previous work [78] and were brought forward in front of the positively charged electrode and the negatively charged electrode, respectively. A non-conductive spacer was inserted into the 1 mm gap that existed between the membranes in order to both stop electrical short-circuiting and creates the flow channel. To maintain a consistent electrical voltage throughout the device, a DC power supply was used. In order to regulate the volume of the feed’s flow, a peristaltic pump was used. A data logger (DT 85) sent the data that were measured by conductivity and pH sensors to a computer that was in a laboratory. Figure 2 contains a diagrammatic representation of the MCDI set-up as well as all the layers that are included inside the MCDI cell for batch and SP modes of operation. The process of adsorption was carried out at a consistent electrical voltage of 1.4 V, whereas the process of desorption took place at a voltage of zero in CDI and a voltage with the opposite polarity of 1.4 V in MCDI. Each experiment was carried out not once, but twice, and the findings that are reported here are the culmination of an adequate number of absorption and desorption cycles that were carried out in order to guarantee consistent behaviour between each cycle.

### 2.6. Experimental Procedure

Experiments conducted using MCDI took advantage of two distinct modes of operation. The salt solution was continually supplied into the flow channel at a flow rate of 60 mL/min from a 1500 mL recycling reservoir, and it was returned to the same reservoir, for a period of 15 min, while the batch mode operation was being carried out (Figure 2a). In order to track the movement of the salt concentrations over time, a conductivity probe was submerged in the reservoir. The electrodes were subjected to a steady electrical voltage of 1.4 volts while the adsorption stage was being carried out. During the single-pass mode of operation (Figure 2b), the feed solution was allowed to go through the cell at a flow rate of 30 mL/min while the conductivity of the effluent was measured. Both the charge and discharge voltages that were used were identical. After the desorption process was finished, the MCDI unit underwent a brief air flush, which removed any remaining brine. As a result, there was no change in the standard of the water that was generated throughout the adsorption phases. We were able to determine the NaCl concentration of the effluent by making use of the connection that existed between the NaCl content and the conductivity.

### 2.7. Evaluation of the Efficiency and Performance of the Capacitive Deionization

The desalination performance of CDI-based technologies is inextricably linked to the operating circumstances of the device, including the operation mode (current or voltage constant mode), flow rate, and composition of the feed stream, in addition to the cell characteristics [79]. Because of this, the performance of these technologies has been represented in a variety of various ways, such as removal efficiency and rate and salt adsorption capacity, as well as charge efficiency and energy consumption (Equations (8)–(13)) [80,81,82]. The salt removal efficiency, often known as SRE, is denoted by the formula of Equation (6) [80,81,82,83]. The salt removal efficiency, often known as SRE [84], is defined as follows:

(8) $$\%SRE=\frac{\left(C_{o}-C_{F}\right)}{C_{o}}100$$

where $C_{o}$ and $C_{F}$ are the starting and final feed water salt concentrations in mg/L. The salt adsorption rate (SAR) is the ratio of SAC by the operation time t, whereas the salt adsorption capacity (SAC) is the total mass of ions removed by the total mass of electrodes (m) during the sorption stage [85,86].

(9) $$SAC\left(mg/g\right)=\frac{\left(C_{o}-C_{F}\right)V}{m}$$

(10) $$SAR\left(mg/g.s\right)=\frac{SAC}{t}=\frac{\left(C_{o}-C_{F}\right)V}{mt}$$

V is feed water treated. The average salt adsorption rate (ASAR) is provided in terms of electrode effective area ($A_{E}$) [79]:

(11) $$ASAR\left(mg/{cm}^{2}.s\right)=\frac{\left(C_{o}-C_{F}\right)V}{A_{E}t}$$

The charge efficiency (CE) reveals how much electric charge is utilised to remove ions, which affects energy consumption and parasitic processes. Thus:

(12) $$\%CE=\frac{\left(C_{o}-C_{F}\right)VF}{M\int_{0}^{t}Idt}$$

where I is the electric current (A), F is the Faraday constant (96,485 C/mol), and M is the molar mass [87], commonly NaCl (58.44 g/mol) [88]. Fang et al. [87] discuss the constant voltage operation mode’s EEC:

(13) $$EEC\left(J/mg\right)=\frac{E\int_{0}^{t}tdt}{\left(C_{o}-C_{F}\right)V}$$

## 3. Results and Discussions

### 3.1. Ion-Exchange Membranes Characterisations

#### 3.1.1. Characterization of Fabricated Cellulose Acetate IEMs vs. Other Membranes Used in MCDI

The initial investigations of ion-exchange materials for membrane capacitive deionization (MCDI) encompassed the utilization of cation-exchange membranes produced from copolymers derived from sodium methacrylate (NaSS), methacrylic acid (MAA), and methyl methacrylate (MMA) (NaSS-MAA-MMA). A membrane with anion-exchange properties was fabricated using the copolymer vinylbenzyl chloride-co-ethyl methacrylate-co-styrene (VBC-EMA-St) [37,89] Similarly, sulfonated composite electrolyte membranes (CEMs) were synthesised by subjecting various copolymers to concentrated sulfuric acid, subsequently followed by solution casting and phase inversion techniques. (106) The aforementioned studies successfully manipulated material properties, including IEC, area resistance, and water absorption, through the implementation of MCDI. Nevertheless, although the membrane exhibited favourable properties, its desalination performance was not thoroughly evaluated using the MCDI performance metrics described earlier. The performance of MCDI was assessed by quantifying the charge–discharge current, which serves as an indicator of the formation of electrical double layers at the electrode surface, a phenomenon that takes place in (M)CDI. The absence of established performance metrics for the MCDI during the time of these studies (Table 2 and Table 3) could have contributed to this outcome. The AEM and CEM-obtained characterization results with respect to IEC, and % swelling and thickness were very close to the results obtained from the cited literature presented in Table 2 and Table 3.

#### 3.1.2. Fourier Transform Infrared (FTIR)

**Figure 3:**
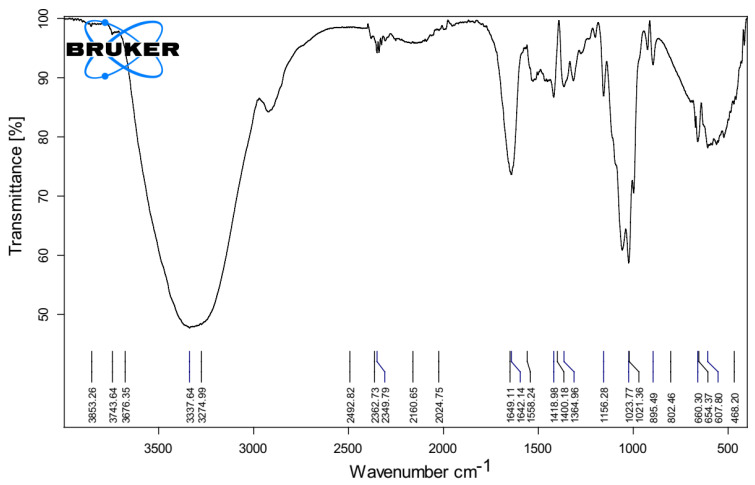
The Fourier transform infrared (FTIR) spectra of the membrane fabricated using cellulose acetate (CA) material.

The Fourier transform infrared (FTIR) spectra of the membrane that was fabricated, as depicted in Figure 3, indicate the presence of an OH functional group within the range of 3000–3750 m^−1^. In addition to the conventional hydroxyl group, which imparts super-hydrophilicity to the membrane by virtue of three hydroxyl (OH) groups per anhydro-glucose unit, the supplementary peak observed at 1640–1630 m^−1^ elucidates the process of water adsorption. This peak facilitates the disruption and formation of hydrogen bonds in water, thereby promoting rapid water permeation across the membrane. In addition, the observed peak at 1125–1170 m^−1^ can be attributed to the asymmetric stretching vibration of the C–O–C bond in the arabinose side chain. Similarly, the peak at 1040–1050 m^−1^ corresponds to the stretching of the C–O bond in the C–O–C glycosidic bonds, providing evidence for the existence of ether linkages between the anhydro-glucose units and the asymmetric stretching of the arabinose side chain. Furthermore, it was determined that none of the solvents or additives were retained within the membrane matrix and subsequently released during the processes of coagulation and post-washing. The Fourier transform infrared (FTIR) spectra analysis revealed that the addition of ion exchange resin (IER) did not result in any notable alterations to the structure of the membrane.

#### 3.1.3. Contact Angles

There is a prevalent assumption that contact angles serve as an indicator of a surface’s hydrophilicity, denoting the degree of attraction between liquid and solid molecules. When the liquid molecules exhibit a strong affinity for the solid molecules, the liquid droplet will uniformly disperse across the solid surface, resulting in a contact angle of 0°. In this particular instance, the surface exhibits super-hydrophilicity, similar to the membranes we utilized, which possessed a contact angle of 13. Consequently, the outcome underscores the super-hydrophilic nature of our membrane.

### 3.2. Changes in Treated Water Conductivity with Cell Potential (Single Pass MCDI)

**Figure 4:**
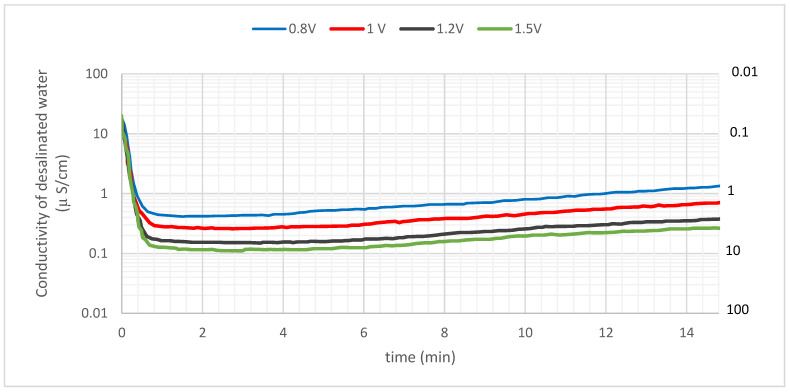
The effluent’s conductivity (or resistance) as a function of time for different cell potentials with a 10 mg/L NaCl feed concentration.

Experiments on desalination were carried out by adjusting the cell voltage from 0.8 to 1.5 V while maintaining the feed concentration of NaCl at 10 mg/L. Figure 4 illustrates the conductivity of the treated water as a function of the potential. On the secondary y-axis, there is also a measurement for electrical resistivity, which is the opposite of conductivity for the sake of comparison.

The conductivity of the influent solution was 20.2 µS/cm, which corresponds to an initial NaCl concentration of 10 mg/L. As the solution passed through the MCDI cell, the conductivity dropped dramatically and reached its lowest value after about 40 s. This is due to the fact that the MCDI cell is a membrane-based electrochemical process that uses an electric field to drive the transport of ions through a semi-permeable membrane. The ions are then adsorbed onto the carbon electrodes, which reduces the conductivity of the effluent. The lowest value of the conductivity of the effluent was lowered to 0.11–0.42 µS/cm when the cell potential was between 0.8 and 1.5 V. This corresponds to an electrical resistivity of between 2.4 and 9.1 MΩ cm. The quantity of adsorption was calculated based on the conductivity of the effluent, which revealed that the average salt removal effectiveness over 900 s (15 min) ranged from 96.3 to 99.1% depending on the cell potential. These results show that the MCDI cell is an effective method for removing salt from water. The salt removal effectiveness is high, and the process is relatively fast. The MCDI cell could be used to treat a variety of water sources, including seawater, wastewater, and brackish water.

The production process for semiconductors requires ultrapure water (UPW) with a resistivity of 18 ΩM cm, while the pharmaceutical and medical sectors employ UPW with a resistivity of roughly 1–10 ΩM cm. As can be observed in Figure 3, the electrical resistivity of the effluent remained higher than 1 ΩM cm at every cell potential that was tested. In particular, the findings point to the fact that a UPW of around 10 M Ω cm may be achieved by applying a potential of 1.5 V. The standard MCDI technique with carbon electrodes operates at a voltage of around 1.2 V [98,99,100]. Based on these findings, we concluded that the MCDI technology makes it possible to readily generate UPW of the quality that is needed by several businesses.

### 3.3. Batch vs. Single-Pass Mode of Operation

**Figure 5:**
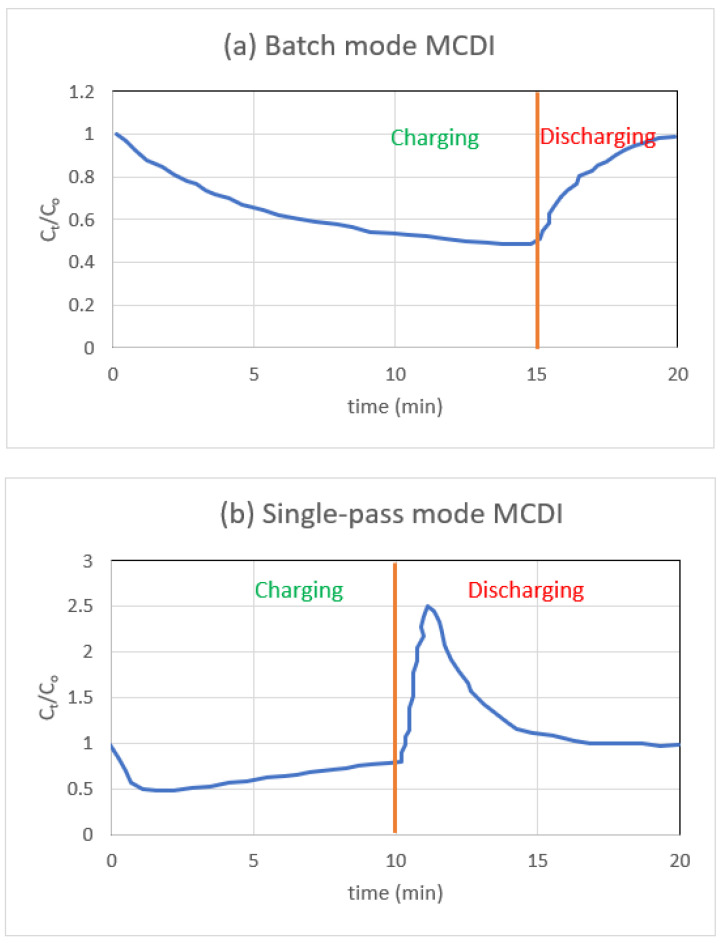
NaCl solution molecular concentration (**a**) as a function of time during batch-mode MCDI relative to the starting concentration (10 mM) in the recycling reservoir, and (**b**) as a function of time during single-pass mode MCDI relative to the feed concentration (10 mM).

Figure 5a demonstrates the usual concentration shift that occurs in the recycling reservoir over one complete cycle of batch mode adsorption and desorption, and Figure 5b illustrates the salt concentration that is present in the effluent stream when the single-pass mode of operation is in use.

As part of the single pass technique, water is pumped from the reservoir into the MCDI cell, and the conductivity is measured as soon as the water leaves the cell. After applying a voltage, the conductivity of the sample, which represents the concentration of salt, will initially reach its lowest possible point for single-pass measurements. The electrodes eventually approach saturation, which causes ions to be expelled from the electrodes which, in turn, causes the conductivity to return to the concentration of the influent. A significant amount of feed solution is needed for any SP experiments you want to do. This prevents the salt content in the influent stream from being subject to significant swings in either direction.

Experiments using batch mode test the conductivity of the feed reservoir, which may be a much lower volume than the reservoir measured using SP mode. When conducting batch mode measurements, the capacity of the feed reservoir must be kept to a minimum because, otherwise, the conductivity changes that occur are much too minute to be recorded correctly. Experiments performed by batch mode reveal that conductivity gradually decreases as time passes. When the electrode reaches its saturation point, the conductivity will settle down to its lowest possible value (maximum salt adsorption). The number of ions that were eliminated may be calculated by comparing the levels of conductivity at the beginning and the end of the process. While batch mode has the benefit of it being easier to operate and analyse the data, a single pass is more reflective of industrial MCDI due to the fact that the feed is not continually recycled, and the effluent is collected [81].

### 3.4. CDI vs. MCDI (Single-Pass Mode)

**Figure 6:**
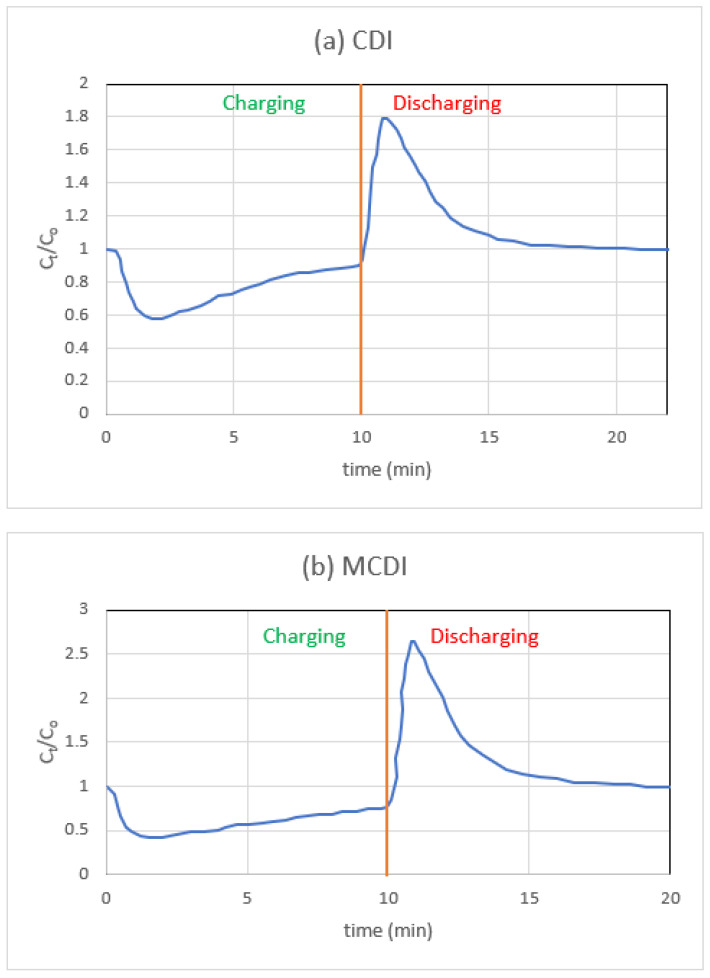
Experimentally determined shifts in effluent salt content after NaCl treatment for (**a**) CDI and (**b**) MCDI cells.

Figure 6a depicts the fluctuation in CDI concentration across a single cycle. Looking at Figure 4a more closely, we see that there is a little rise at the start of each adsorption phase. Since the voltage during regeneration is zero, an undesirable amount of co-ion adsorption will occur during this desorption process. Therefore, a sudden repulsion of these co-ions occurs when reapplying the cell voltage during the second adsorption stage, leading to a repulsion peak [48].

In the subsequent set of tests, an MCDI configuration was created by positioning the fabricated IEMs in front of the electrodes (Figure 6b). In these studies, the length of adsorption was maintained at a level comparable to that of CDI; however, the needed time for desorption was reduced, since zero voltage was replaced with a polarity that was inverted. When employing IEMs, there are fewer variations in the concentrations of the various ions, and the charge efficiency is substantially greater, as other writers [47,48] have observed. According to Biesheuvel et al. [54] and Gao et al. [55], the repulsion peak that was present at the beginning of the adsorption stage is no longer present. This confirms that the peak detected in CDI results from co-ion repulsion and does not result from immobile chemical charges. In the MCDI cell, it is evident that the influence of hydrated radii is no longer significant for NaCl monovalent ions, which suggests that the IEMs themselves are now regulating the mass transfer. The normalised charge efficiency of MCDI is, as a matter of fact, consistently high. When using MCDI, this increase is brought about as a result of co-ion blocking and regeneration at reversed voltage. The counter-ions, which are ions that have an opposing charge to the electrode during adsorption, not only leave the micropores of the carbon electrodes when the polarity is reversed but the macropores also become depleted. This happens because the counter-ions are attracted to the opposite charge. Because of this, during the subsequent stage of adsorption, there is a greater pushing force for the ions of opposite charge to permeate through the macropores and adsorb into the EDLs contained inside the micropores. On the other hand, in CDI, when the voltage goes to zero, the ions that have been adsorbed are repelled; nonetheless, their concentration in the macropores of the carbon merely reduces to that of the bulk stream. This is because CDI does not have an electrostatic field [101].

### 3.5. Effect of pH

**Figure 7:**
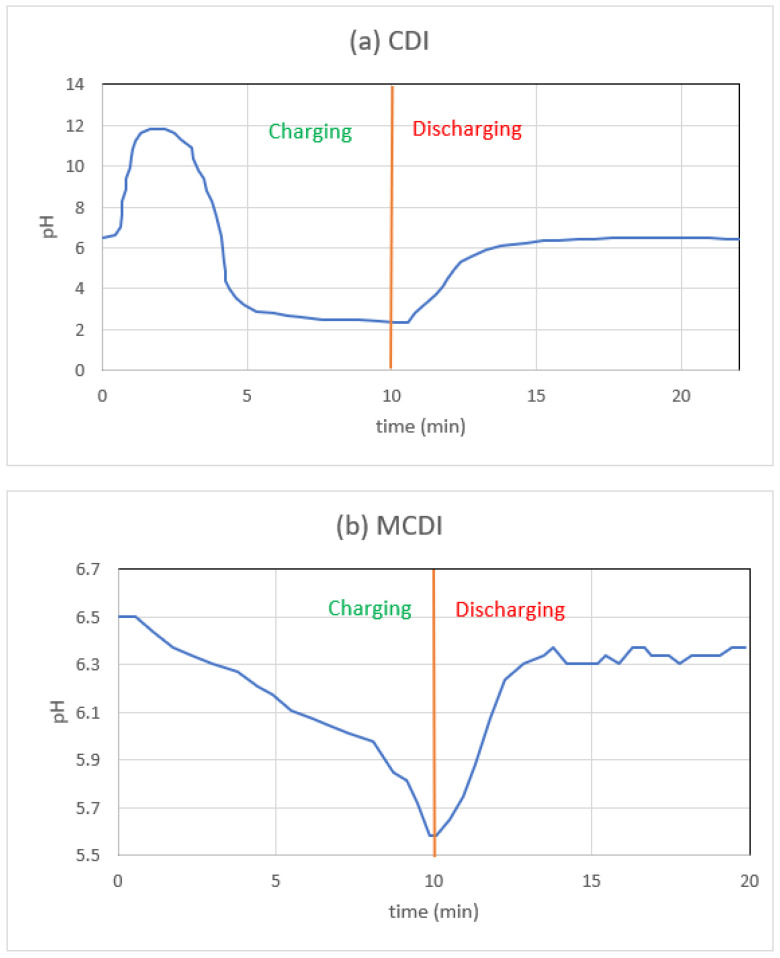
One adsorption–desorption cycle’s worth of MCDI and CDI effluent pH readings from studies with NaCl.

Figure 7a,b shows the single-pass CDI and MCDI effluent pH variation for NaCl electrolytes. All desalination studies begin with a feed solution of pH 6.5, but the initial effluent pH varies depending on the number of treatment cycles that came before it. The pH changes from high to low during the adsorption of NaCl salt solution in the CDI arrangement (Figure 7a). Faradaic reactions (oxidation-reduction) and ion electrosorption (a non-faradaic effect) both contribute to the pH swing [50]. In industrial applications, a sudden shift in pH can cause unwanted salt precipitation and electrode fouling. Above 1.0 V in the cell, Lee et al. [100] found a similar pH trend.

While the pH shift during salt adsorption in CDI appears significant, with a peak of 11.6 and a minimum of 2.3, the change in H^+^ concentration is restricted to only 0.3 mM. Compared to the observed shifts in salt concentration, this is extremely minor. Therefore, the measurements of salt concentration are unaffected by the shifts in H^+^ concentration. The relatively high voltage (1.4 V) utilized in these studies may potentially contribute to the considerable pH variation. While less salt would be removed by operating at a lower voltage, we recommend doing so in processes that are sensitive to such shifts in pH.

However, as shown in Figure 7b, MCDI’s pH change range is narrower than that of CDI, especially during the early increase. The reasons for this could be a variety of things. When the CEM is positioned in front of the cathode, dissolved oxygen is blocked from reaching the carbon surface. The recent paper by Tang et al. [51] makes use of this same line of reasoning. Secondly, even if some oxygen does make it to the cathode, the OH^−^ that is created in the process has a hard time crossing the CEM and entering the bulk solution. Thus, reaction redox potentials are not affected by the absence of the initial pH jump. Because of the AEM, the generated H^+^ ions have a hard time making their way into the bulk stream. We further postulate that hydroxyl and hydronium ions, if they are indeed trapped behind each ion-exchange membrane, can boost the counter-ion attraction.

## 4. Conclusions

Because water shortage is widely acknowledged to be the most significant environmental concern of the 21st century, desalination continues to be the most effective way to make up for lost supplies of clean water. However, many desalination devices need a significant amount of electricity, which is often in short supply in nations with low per capita incomes. The use of capacitive deionization to produce clean water seems promising for a number of reasons, including the capacity to run the process at a low potential and with a low amount of energy, the availability of vast quantities of agricultural wastes for use in the synthesis of electrodes, IEM and so on. Producing ultrapure water with CDI is a process that is both efficient and kind to the environment. This technology is continually being enhanced by the addition of new chemicals and membrane materials, which might have huge benefits throughout the globe in terms of both the environment and the economy. When compared to alternative physical methods for the purification of diluted feeds or molecular separations, CDI is a process that is both non-destructive and electrokinetically based. It also has a very high energy efficiency rating. The CDI system has several other fascinating qualities, such as its adaptability, small size, reduced generation of secondary chemical waste, molecular selectivity, and propensity to link separations and reactions. The use of IEM allowed MCDI to significantly decrease the co-ion effect, which resulted in an improvement in the performance of the desalination process.

- This study demonstrates that the deacetylated heterogeneous IEMs may be simply manufactured using the phase inversion approach.
- When IEMs were placed in front of the electrodes, there was a discernible increase in both the charge efficiency and the salt adsorption rates.
- Since a sudden increase or reduction in pH is not desirable for the generation of fresh water, MCDI, as opposed to CDI, only slightly alters the pH of the water. This is an additional advantage of MCDI over CDI.

MCDI outperforms CDI in salt removal, energy efficiency, and cost. MCDI requires additional maintenance and has a lower fouling tolerance. MCDI, a cutting-edge desalination method, has significant advantages over CDI. However, MCDI is currently in development and not commercially accessible. MCDI, a revolutionary technology, might transform the desalination business. MCDI might generate clean water for a rising population, at a low cost and sustainably, with more study.

Lastly, CDI/MCDI can help the desalination industry because they use less energy and work at lower pressures than traditional methods like RO. Their scalability also makes them good for a number of applications, such as decentralised desalination systems and point-of-use water purification, brackish water desalination where the salt concentration is lower than in seawater, and pre-treatment for RO by removing a large number of ions and organic matter. CDI/MCDI can enhance the efficiency and lifespan of RO membranes, remove specific contaminants by adjusting the properties of the IEMs and optimising the operation conditions, target specific pollutants, such as heavy metals, nitrates, or organic compounds, regenerability and reusability reduce the environmental impact and operational costs associated with desalination, and integration with renewable energy sources enables sustainable and eco-friendly desalination, reducing reliance on fossil fuels and mitigating the environmental impact.

Although CDI/MCDI continues to encounter obstacles related to efficiency, cost, and membrane durability, current research and technological progress are focused on overcoming these limitations and improving the role of CDI/MCDI in the domain of desalination.

## Figures and Tables

**Table 1:** Comparison between the energy consumption of several desalination methods.

Desalination Technology	Driving Force	Energy Consumption (kWh/m^3^)	Cost of Water USD/m^3^	Refs.
MSF	Thermally driven	5–6	0.56–1.75	[69]
MED		3–4	0.52–8	[70]
ED	Potential difference	2.6–5.5	0.6–1.05	[71]
Seawater RO	Pressure	5.1–7.45	0.45–1.72	[72]
Brackish Water RO		1	0.26–1.3	[73]
CDI	Potential difference	0.1–1.5	0.11	[68,74]

**Table 2:** The present analysis focuses on the membrane properties of anion-exchange membranes that have been specifically designed for use in membrane capacitive deionization (MCDI) systems.

Membrane Type	Characterizations					Ref.
	Thickness (μm)	% Swelling	% Perm-Selectivity	IEC, mmol eq/g	Electrical Resistance (Ω cm^2^)	
VBC-EMA-St	–	23–68		0.9–1.7	1.6–8.8	[89]
QPVA	–	7–31		1.2–2.8	–	[90]
PVDF-*g*-VBC	–	12–56		0.4–1.3	2.0–15	[91]
PE-CMS	29	5		3.0	0.3	[92]
QPPO	62–70	10–90		1.3–2.6	1.0–9.0	[93]
CA-AEM	0.581	70.87	37	1.34	5	This study

**Table 3:** The present analysis focuses on the membrane properties of cation-exchange membranes that have been specifically designed for use in membrane capacitive deionization (MCDI) systems.

Membrane Type	Characterizations					Ref.
	Thickness (μm)	% Swelling	% Perm-Selectivity	IEC, mmol eq/g	Electrical Resistance (Ω cm^2^)	
VBC-EMA-St	–	76–121		0.5–1.0	0.6–2.8	[89]
PE-CSPS	25	26–36		0.7–1.0	0.3–0.6	[94]
PVDF-*g*-PSVBS	–	7–61		0.1–1.1	2–60	[95]
sPEEK	100	28–47		2.0–2.3	0.5–0.9	[96]
sPBC	–	40–200		1.0–2.0	–	[97]
CA-CEM	0.526	74.28	44.88	1.47	5	This study

## Data Availability

Not applicable.
